# Emotions explain differences in the diffusion of true vs. false social media rumors

**DOI:** 10.1038/s41598-021-01813-2

**Published:** 2021-11-22

**Authors:** Nicolas Pröllochs, Dominik Bär, Stefan Feuerriegel

**Affiliations:** 1 JLU Giessen, Giessen, 35394 Germany; 2 LMU Munich, Munich, 80539 Germany; 3 ETH Zurich, Zurich, 8092 Switzerland

**Keywords:** Human behaviour, Information technology

## Abstract

False rumors (often termed “fake news”) on social media pose a significant threat to modern societies. However, potential reasons for the widespread diffusion of false rumors have been underexplored. In this work, we analyze whether sentiment words, as well as different emotional words, in social media content explain differences in the spread of true vs. false rumors. For this purpose, we collected ${\varvec{N}} =126{,}301$ rumor cascades from Twitter, comprising more than 4.5 million retweets that have been fact-checked for veracity. We then categorized the language in social media content to (1) sentiment (i.e., positive vs. negative) and (2) eight basic emotions (i. e., anger, anticipation, disgust, fear, joy, trust, sadness, and surprise). We find that sentiment and basic emotions explain differences in the structural properties of true vs. false rumor cascades. False rumors (as compared to true rumors) are more likely to go viral if they convey a higher proportion of terms associated with a positive sentiment. Further, false rumors are viral when embedding emotional words classified as trust, anticipation, or anger. All else being equal, false rumors conveying one standard deviation more positive sentiment have a 37.58% longer lifetime and reach 61.44% more users. Our findings offer insights into how true vs. false rumors spread and highlight the importance of managing emotions in social media content.

## Introduction

A vast number of social media users have been exposed to knowingly false content. This was confirmed to be the case during humanitarian crises^1^ and elections^2–5^. For example, in the 2016 U. S. presidential election, each adult was shown, on average, more than one item with false content^6^. On top of that, there were more user interactions with deliberately false content than with reliable information sources^7^. To this end, false content on social media poses a threat to individuals, organizations, and even whole societies^8,9^.

Understanding the spread of false content is of wide interest^2,9^. For users, understanding this phenomenon could yield certain signals based on which true and false content can be recognized. For social media platforms, a better understanding could inform the design of early warning systems that automatically detect the spread of false content^10^. Specifically, it would allow one to derive features from the propagation dynamics of false content that could then be fed into machine learning classifiers^11–14^. For policy makers, understanding the spread of false content is necessary for developing mitigation strategies that directly target the viral effects of false content (e. g., educating users to exercise more critical thinking when confronted with emotional content). This is especially critical as repeated exposure to false information has led many users to erroneously believe that it was true^15^.

Only a few studies have focused on understanding differences in the spread of true vs. false social media content. True vs. false rumors have been compared across different characteristics of resharing cascades by Refs.^16,17^. They observed larger, wider, and deeper cascades for false rumors. Further, some emotions are more often found in false rumors^18^; however, it does not link emotions to differences in diffusion across true vs. false rumors.

In this work, we hypothesize that differences in the diffusion of true vs. false rumors can be explained by the conveyed sentiment and basic emotions. Our rationale is motivated by prior literature. Emotions are highly influential for human judgment and decision making^19^, and strongly affect how humans draw or capture attention^20^. Emotions are highly contagious and thus spread through direct interaction within a social network^21–23^. Emotions have also been found to impact retweeting^24^, thus driving diffusion^21,25,26^. To this end, emotional stimuli trigger cognitive processing^27^, which in turn results in the behavioral response of information sharing^28–30^. Reliance on emotions further promotes belief in false information^31^. Altogether, this suggests that sentiment and emotions might offer a potential explanation for differences in the spreading dynamics of true vs. false rumors; however, empirical evidence is lacking.

Prior literature has established sentiment, as well as emotions, to be drivers of online diffusion^24,26,32–37^. However, these works suggest that their roles regarding different types of online content vary. For example, the spreading of news has been found to be promoted by positive sentiment^26,34^, whereas the diffusion of health-related content is driven by negative sentiment^35^. Another work studies how sentiment promotes the diffusion of online rumors^38^. However, the sample used in this study only comprises rumors for a single crisis event, thus motivating us to analyze the role of sentiment and emotions in the spreading of true vs. false rumors.

We perform a large-scale explanatory analysis from observational data and, based on this, quantify to what extent language characterized by sentiment and basic emotions explain cascades of true vs. false rumors (see “Materials and methods”). We focus our analysis on three common structural properties of cascades: (1) size, (2) lifetime, and (3) the so-called “structural virality”^39^. These metrics quantify (1) how many users they reach, (2) how long rumors persist, and (3) how effectively they spread through the social network (i. e., a breadth-depth trade-off^39^).

Using a text mining framework, we extract sentiment and emotions embedded in replies to rumor cascades according to Plutchik’s emotion model^40^. Plutchik’s emotion model provides a comprehensive categorization across 8 basic emotions (i. e., anger, anticipation, joy, trust, fear, surprise, sadness, and disgust) that are regarded as universally recognized across cultures^41,42^. We compute a sentiment score that measures the overall valence of the text, that is, whether words are categorized more often as positive or negative. We then use hierarchical generalized linear models with one-way interactions in order to capture differences in the effects of sentiment and basic emotions across veracity. Here we control for between-rumor heterogeneity, specifically the social influence of senders (e. g., we correct for the number of followers, etc.).

To address our research questions, we analyze $N = 126{,}301$ rumor cascades from Twitter. Our data provides a large-scale, cross-sectional sample based on a comprehensive set of cascades on Twitter during the time period from the founding of Twitter in 2006 through 2017. In particular, our sample contains all English-language tweets that were subject to fact-checking by one of five different fact-checking organizations (see “Materials and methods”). Overall, this amounts to $\sim 4.5 \,{\text {million}}$ retweets by $\sim 3 \,{\text {million}}$ different users.

In summary, we study whether variations in language characterized as (1) positive and negative sentiment and (2) certain emotions (e. g., anger, anticipation, trust) explain differences in the structural properties of true vs. false rumor cascades on social media. For this, we draw upon a large-scale dataset of true and false rumors from Twitter and, on this basis, analyze the effect across a comprehensive, fine-grained set of emotions.

## Results

**Figure 1:**
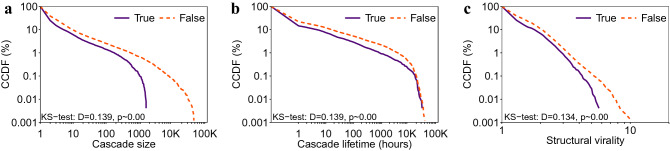
Complementary cumulative distribution functions (CCDFs) for different diffusion properties of social media cascades, namely, cascade size (**a**), cascade lifetime (**b**), and structural virality (**c**).

Cascades of true and false rumors exhibit different structural properties. Figure 1 compares the diffusion based on the complementary cumulative distribution functions (CCDF). Overall, we find that false rumors are characterized by cascades of larger size and longer lifetime. For instance, the average cascade lifetime for false rumors is 149.61 h, whereas it is 71.62 h for true rumors. Furthermore, false rumors also entail cascades with higher structural virality.

**Figure 2:**
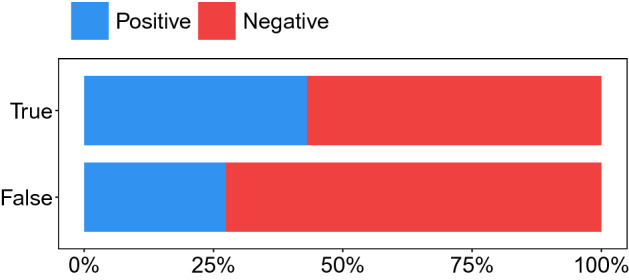
Relative frequency of true and false rumor cascades associated with positive vs. negative language.

**Figure 3:**
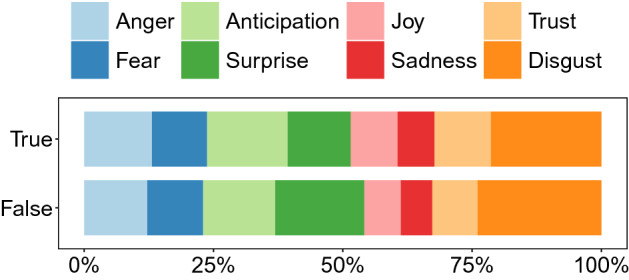
Average emotion score in true and false rumor cascades, following Plutchik’s wheel of emotions^40^.

**Figure 4:**
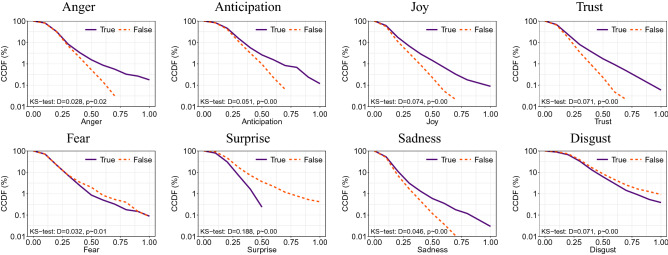
Complementary cumulative distribution functions (CCDFs) for conveyed emotions. Statistical comparisons are based on a Kolmogorov–Smirnov (KS) test.

True and false rumors also convey language of different sentiment and with different emotions. As shown in Fig. 2, the language in false rumors is more often associated with negative sentiment than in true rumors. In addition, Fig. 3 shows that false rumors convey a higher proportion of words classified as disgust, fear, and surprise, while true rumors are more likely to be linked to anger, anticipation, joy, sadness, and trust. In Fig. 4, we plot the CCDFs for each of the eight basic emotions. Evidently, false rumors are more likely to contain words associated with fear, disgust, and surprise, whereas true rumors contain words associated with sadness but also anger, anticipation, joy, and trust. Kolmogorov-Smirnov (KS) tests confirm that these differences are statistically significant.

### Analysis of sentiment

**Figure 5:**
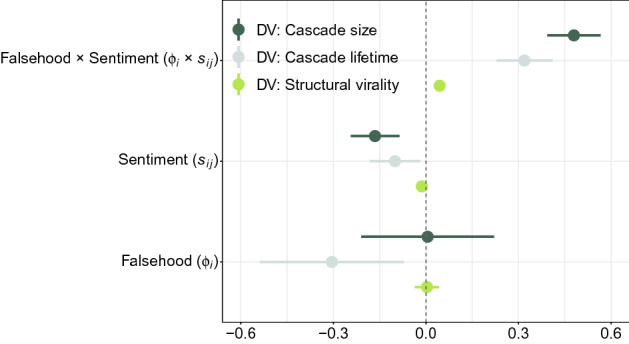
Standardized parameter estimates and 95% confidence intervals.

We fit explanatory regression models to evaluate how variations in sentiment (i. e., the difference between positive vs. negative word counts) are associated with differences in the structural properties of true vs. false rumor cascades (see “Materials and methods” and Supplementary Table S1). In Fig. 5, the parameter estimates establish a pronounced role of sentiment ($s_{ij}$) with significantly different estimates for true vs. false rumors. For each dependent variable (DV), we observe negative coefficients for the sentiment variable, meaning that true rumors diffuse more pronouncedly if negative language is present. The positive coefficient for the interaction term (${ Sentiment } \times { Falsehood }$) suggests the opposite effect for false rumors. Compared to true rumors, a one standard deviation more positive sentiment for false rumors is linked to a 61.44% increase in the cascade size, a 37.58% increase in the cascade lifetime, and a 4.81% increase in structural virality. Notably, the estimated effect sizes are larger for false as compared to true rumors. Hence, positive sentiment appears to promote the diffusion of false rumors (while negative sentiment is estimated to promote the diffusion of true rumors).

**Figure 6:**
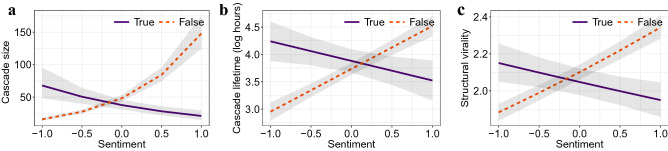
Predicted marginal means of cascade size (**a**), cascade lifetime (**b**), and structural virality (**c**) for different values of the sentiment variable. The 95% confidence intervals are highlighted in gray.

Figure 6 shows the predicted marginal mean effect of the sentiment variable on the DVs. For each DV, we find relatively large effect sizes for the sentiment variable that significantly differ between true vs. false rumors. All else being equal, false rumors have cascades that are of larger size, longer duration, and greater virality if the sentiment is positive. Hence, a (positive) sentiment in the language of rumors explains the pronounced diffusion of false rumors.

Our regression model controls for heterogeneity in users’ social influence (see Supplementary Table S1). In short, rumor cascades initiated from accounts that are verified and younger are linked to a larger, longer, and more viral spread. Similar relationships are observed for users exhibiting greater numbers of followers and followees. In contrast, a higher engagement level is negatively associated with the size, lifetime, and structural virality of a cascade.

We calculated the pseudo-$R^2$ for each model, resulting in relatively high values of 0.64 for cascade size, 0.43 for cascade lifetime, and 0.31 for structural virality. Evidently, the model variables explain a large proportion of the DV variations. Furthermore, visual inspection of the actual vs. fitted plot and goodness-of-fit tests indicate that the models are well specified. This is also supported when considering the differences between the AIC models for individual models estimated with/without sentiment variables. For each DV, the difference is greater than 10 (cascade size: 303.43; lifetime: 110.56; structural virality: 170.01), indicating strong support for the corresponding candidate models^43^. Therefore, the inclusion of sentiment variables in the regression model is to be preferred.

### Analysis of emotions

Plutchik’s emotion model arranges the eight basic emotions into four pairs of bipolar emotions (see “Materials and methods”). We now evaluate how these bipolar emotion pairs are associated with differences in the structural properties of true vs. false rumor cascades (see coefficient estimates in Supplementary Table S2). The reason for using bipolar emotions is the strong linear dependence among the 8 basic emotions. Adding all basic emotions to the same model would make the estimation rank-deficient. As a remedy, we focus on bipolar emotions, which allow for all eight basic emotions to be examined in the same model.

**Figure 7:**
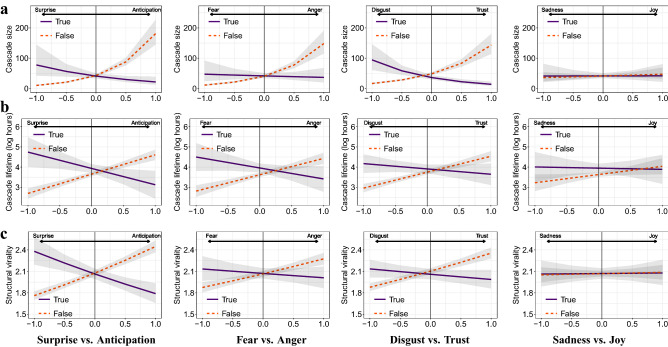
Predicted marginal effect of language classified by bipolar emotions on cascade size (**a**), cascade lifetime (**b**), and structural virality (**c**). The 95% confidence intervals are highlighted in gray.

The predicted marginal effects for the bipolar emotion pairs are shown in Fig. 7. Changes in the emotional language dimensions are associated with greater changes in size, lifetime, and structural virality for false rumors vs. true rumors, as evidenced by steeper slopes of the curves. We observe that false rumor cascades containing words associated with anticipation, anger, and trust have a more extensive diffusion than their true counterparts. We find no statistically significant coefficient for language related to joy vs. sadness. In summary, false rumors spread more extensively than true rumors in the presence of emotional language embedding anticipation, anger, and trust, whereas we observe opposite effects, albeit of smaller magnitude, for language connected to surprise, fear, and disgust.

## Discussion

Here we analyze to what extent language embedded in online content can explain differences in the spread of true vs. false social media rumors. Specifically, we study two dimensions: (1) sentiment and (2) basic emotions. Our results establish that both are important determinants of the different spread of true vs. false rumors. For sentiment, we find that positive language is associated with a wider, longer, and more viral spread for false rumors. For basic emotions, we find that language characterized as anger, anticipation, and trust is associated with a wider, longer, and more viral spread for false rumors.

Our research is based on the following rationale as to why sentiment (and emotions) should have the ability to influence the spread of true vs. false rumors. Sentiment (and emotions) are highly relevant for diffusion of online content^24,26,34–36,44,45^. For instance, prior research has studied the role of sentiment in the diffusion of online rumors during crisis^38^. Similarly, online rumors are characterized by a distinctive set of emotions^17^. Hence, this motivated our research to examine whether sentiment (and emotions) are determinants for the distinct spread of true vs. false rumors. Different from previous works, we demonstrate that language in the form of sentiment and emotions can explain the unique structural properties of false rumors.

In our research, we studied the role of different discrete emotions (e. g., anger) in promoting the spread of true vs. false rumors. This choice was made for two reasons. First, discrete emotions are commonly used in affective computing. Specifically, we build upon the NRC emotion lexicon which provides a prominent and comprehensive dictionary for examining discrete emotions^46^. This choice renders our analysis comparable to other research. Second, and more importantly, discrete emotions such as anger have been identified as being relevant for offline rumors^47,48^ and online rumors^17,18,37^. Because of this, our analysis also involves discrete emotions. Future research could expand our work and follow a physiological constructionist perceptive as an alternative emotion model (where emotions form a $2 \times 2$ dimensional space around valence-arousal).

This study is subject to the typical limitations inherent in observational inferences. First, we report associations and refrain from making causal claims. Other studies^18^ argue that estimates should resemble those from causal inferences due to the temporal nature whereby the tweet precedes the cascade formation. Second, our inferences are limited by the accuracy and availability of fact-checking labels. Possible selection biases might arise from the preferences and processes of the used fact-checking websites (e. g., partisan biases). Reassuringly, the fact-checking websites reveal high pairwise agreement^17^. Third, our objective was to compare true vs. false rumors. Future research might further investigate rumors that cannot be clearly attributed to one of the two fact-checking labels. Fourth, our dictionary approach does not allow us to infer the physiological state of users and whether certain emotions are inspired. Instead, our dictionary approach quantifies the use of language in text. Thus, it is possible that even if rumors embed words associated with positive language, they may still elicit negative emotions in readers. More research is necessary to understand the relationship between expression and elicitation of emotions in online rumors, i. e., author vs. receiver effects^49^. Fifth, our study builds upon Plutchik’s emotion model and does not account for the the extent of emotionality in rumor cascades, i. e., the extent to which emotional words are present at all. Future research might complement our analysis, by distinguishing the roles of total emotionality and emotional valence in rumor diffusion. Sixth, we follow earlier research and quantify online diffusion by extracting the size, lifetime, and structural virality of cascade. Therefore, our unit of analysis is at the cascade level, which is consistent with earlier research^37,39,50–54^. As such, we expect interesting research opportunities by studying the within-cascade diffusion dynamics.

Policy initiatives around the world require social media platforms to limit the spread of false rumors^9^. To detect them early, our findings emphasize the importance of considering variations in positive and negative words as well as emotional language. In machine learning predictions, sentiment and emotions have been employed in comparatively few works^11–14,55^, despite the fact that sentiment and emotions promise benefits in platform-wide settings: they are likely to be more robust against manipulation than other predictors (e. g., content features, for which predictive power is limited if an unseen topic or keyword is encountered). Sentiment and emotions are also available in the early stages of the diffusion, at which point features from the propagation dynamics are scarce (cf. the discussion in^56^). By managing sentiment and emotions in social media content, platforms might develop an effective strategy for reducing the proliferation of false rumors.

## Materials and methods

### Data collection

We analyze a comprehensive dataset with rumor cascades from Twitter^17^. In particular, we examine a sample of English-language cascades on Twitter from its founding in 2006 through 2017. To this end, rumors were matched against established fact-checking websites (see below). Permission to process this dataset for the purpose of our study was granted by Twitter. This ensures a real-world, large-scale sample. Each rumor in our sample involves one or more *rumor cascades*. A rumor has more than one rumor cascade if it exhibits multiple independent retweet chains started by different users but pertaining to the same story/claim. In sum, our data contains $N =126{,}301$ rumor cascades corresponding to 2448 rumors. The rumors were retweeted more than 4.5 million times by around 3 million different users. The rumors in the dataset cover varying topics (e. g., Politics, Business, Natural Disasters), while the largest proportion of rumors are political rumors^17^.

As per terminology, we adopt the definition of *rumors* used in^17^. In this work, rumors refer to content that can be identified as true or false through fact-checking. This definition emerged in the 1940’s in social psychology literature^57,58^, formalizing it as a proposition involving person-to-person propagation but without necessarily being truthful, such that fact-checking can determine the underlying veracity.

Twitter was selected for this study for the following reasons. First, Twitter represents a social media platform with tremendous popularity^59^. In 2019, it counted $\sim 330$ million active users^60^. Second, Twitter is extensively used for news consumption. Twitter is consulted for information on political matters by one in ten U.S. adults^61^. Third, Twitter is regarded as highly influential in the public discourse, especially concerning political matters^5^, in which deceptive content poses a threat to the functioning of societies.

**Figure 8:**
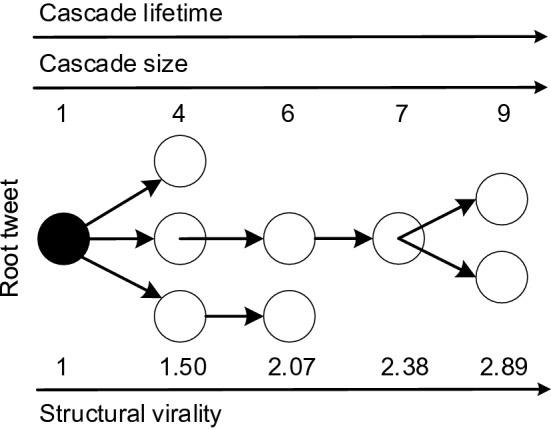
Example tree structure of a rumor cascade.

Our dataset further contains information regarding the retweet path of each rumor cascade, i. e. the temporal propagation dynamics of a rumor cascade on Twitter. Figure 8 shows an exemplary tree structure of a rumor cascade. The root node is the original tweet containing a rumor, whereas the children are retweets of the original tweet and all other nodes are retweets of retweets of the original tweet. We use the retweet path to calculate structural characteristics of each rumor cascade, namely the size (the number of users involved in a cascade), lifetime (the time difference between the root tweet and the terminal tweet), and structural virality.

IRB approval was received from ETH Zurich (2020-N-44). The above data collection results in a large-scale dataset on online rumors.

### Fact-checking

Our data sample comprises a comprehensive set of Twitter cascades that were subject to fact-checking based on at least one of six independent organizations: http://factcheck.org, http://hoax-slayer.com, http://politifact.com, http://snopes.com, http://truthorfiction.com, and http://urbanlegends.about.com. Fact-checking returns labels that denote the veracity of the content according to three categories: true, false, or mixed. Fact-checking websites show high pairwise agreement^17^, ranging between 95 and 98%. True and false labels are even completely disjunct.

In our data, the frequencies of fact-checking labels at cascade level are: 24,409 ($={\text {true}}$) and 82,605 ($={\text {false}}$). For 19,287 rumors, no clear assignment to true or positive was possible; these rumors were discarded in our analysis as we aim at comparing true vs. false rumors. Examples of analyzed rumors are given in Table 1.

**Table 1:** Examples of rumors posted on Twitter. Fact-checking labels from http://politifact.com.

Rumor	Label
*“From 2010 to 2014, median household income has actually gone up 7.4%.”*	FALSE
*“Increasing the min. wage to* $$\$15$$ *an hour would reduce spending on food stamps, public housing and other programs by over* $$\$7.6$$ *billion a year.”*	FALSE
*“Thanks to #ObamaCare, average E.R. wait in California is 5 hours:* http://gop.cm/6015YqKd*And “it’s only going to get worse.””*	FALSE
*“California Gov says yes to poisoning more children with mercury and aluminum in manditory vaccines. This corporate fascist must be stopped.”*	FALSE
*“It’s the longest running congressional investigation ever. It’s cost taxpayers $4 million. And what’s it about?”*	FALSE

Fact-checking labels from the other websites show high pairwise agreement, with true and false labels being completely disjunct^17^.

### Calculation of scores for sentiment and emotions

**Figure 9:**
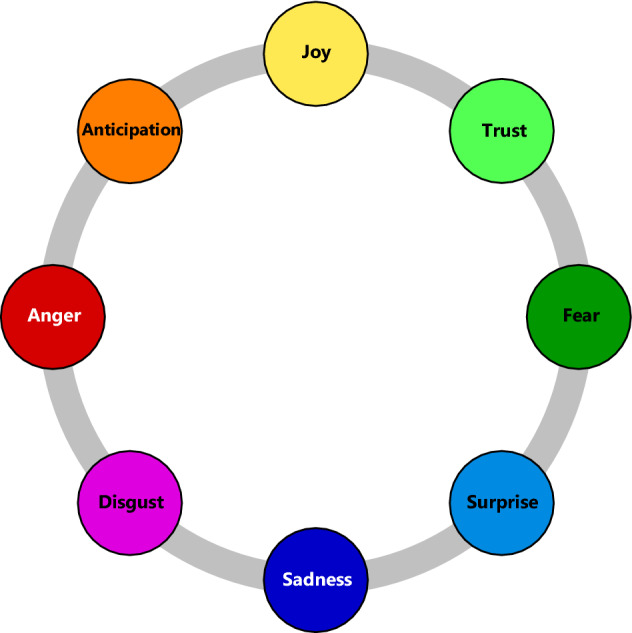
Plutchik’s wheel of emotions^40^.

Scores for sentiment and emotions were computed based on affective computing^62^. Here we use (1) sentiment giving the overall valence across positivity and negativity and (2) eight basic emotions: anger, fear, anticipation, trust, surprise, sadness, joy, and disgust. The basic emotions are defined in Plutchik’s wheel of emotions^40^; see Fig. 9. Basic emotions are rooted in human evolution and are thus stable across ethnic or cultural differences^41,42^. Furthermore, according to emotion theory, basic emotions represent a small subset of core emotion based on which other more complex emotions are derived. As shown in the Plutchik’s wheel of emotions, basic emotions exhibit a bipolar categorization, where each emotion has a corresponding opposite emotion.

The underlying computation of the emotion scores followed the procedure from^17^. For all rumor cascades *j* of rumor *i*, the scores were determined based on the NRC emotion lexicon^46^ that contains a comprehensive list of 141,820 English words and their associations with each of the eight basic emotions. Each reply to the root tweet of a rumor cascade was cleaned, tokenized, and then compared against the NRC emotion lexicon. To illustrate examples of emotional words, Table 2 categorizes a set of online rumors across the eight basic emotions using the NRC emotion lexicon.

**Table 2:** Examples for rumors posted on Twitter and the emotional words they contain.

Emotion	Online rumor
Anger	*“Reports That IS Leader Abu Bakr Al-Baghdadi Was Wounded In A Coalition Air* ***Strike*** *Are “Unconfirmed” - Us Defense Sec John Kirby #R4today”*
	*“Y’all, I Just Read That ABC Paid Darren Wilson $500k For The Interview.* ***Destroying*** *Black Life Remains A Lucrative American Career. #Ferguson”*
Fear	*“Reports Of* ***Shooting*** *At Dammartin En Goele On Route N2 North East Of Paris - French Media Says Car Chase Under Way”*
	*“Sydney* ***Hostage*** *- Taker - Man Monis, 49 - Originally From Iran - Self-Styled Sheikh -****Accused*** *Of Sexually Assaulting 7 Women Developing..”*
Anticipation	*“Obama’s Daughter Is Pregnant LOL Michelle Should’ve Spent More* ***Time*** *With Her Instead Of Taking Away Our French Fries”*
	*“Obama Has Filed Federal Charges Against Zimmerman For Violating Trayvon’s Civil Rights.* ***God*** *Is* ***Good***”
Trust	*“Darren Wilson Is A Six Year* ***Veteran*** *Of The #Ferguson* ***Police*** *And Had No Disciplinary Actions Against Him.”*
	*“Canadian Authorities Have Given Name Of Suspect In Ottawa Attacks To U.S. Feds; Ask For FBI Assistance: Sr U.S.* ***Law*** *Enforcement* ***Official***”
Surprise	*“Breaking: 15.1 Foot Tsunami Reported In Coquimbo.* #**Earthquake** *#Tsunami”*
	*“Walmart Donates $10,000 To Support Darren Wilson, But Won’t Give Tracy Morgan A Penny For The* ***Accident*** *Their Company Caused. #Boycottwalmart”*
Sadness	*“What’s So Frustrating Is That Now We Are Talking About A* ***Robbery*** *And Not The* ***Killing*** *Of An Unarmed Kid. #Ferguson”*
	*“Conservative Caucas Informed* ***Soldier*** ***Shot*** *At War Memorial In Ottawa This Morning Has Died. A Sad Development On A Shocking Day.”*
Joy	*“Disney Are Making Their First Movie To Feature Two Gay Princes Who Fall In* ***Love***, *Amazing.”*
	*“Paula Deen: “Forget The* ***Food*** *Network. I’ve Already Been Offered A Cooking Show On The New Fox News* ***Food*** *Channel.””*
Disgust	*“#Psa Please Do Not Drink Any Pepsi Soda, A Worker From That Company Has Put Blood* ***Contaminated*** *With Aids Inside The Bottles!!! Please Rt!!”*
	*“People* ***Blame*** *The* ***Massacre*** *In Orlando On The NRA. Newsflash: The Orlando Shooter Wasn’t A NRA Member... But He Was A Registered Democrat.”*

The emotional words are classified according to the NRC emotion lexicon using eight basic emotions: anger, fear, anticipation, trust, surprise, sadness, joy, and disgust.

Emotional word corresponding to the basic emotion in column 1.

#### Sentiment

We calculate a sentiment score $s_{ij}$ that only measures the extent of positive/negative polarity in replies to rumor cascades. Based on Plutchik’s wheel of emotions, we compute the word count of all positive words, denoted by ${Positivity} _{ij}$, and the word count of all negative words, denoted by ${Negativity} _{ij}$, respectively. Both scores were normalized so that they add to one, and thus measure the relative extent to which language leans toward a positive or negative polarity. The sentiment score $s_{ij}$ is then defined as the difference between positivity and negativity, i. e., $s_{ij} = {Positivity} _{ij} - {Negativity} _{ij}$.

#### Bipolar emotion pairs

We start by computing the fraction of words in the reply tweets that relate to each of the eight emotions. These were then aggregated and averaged to create a vector of emotion weights that sum to one across the emotions. The eight emotion dimensions in $e_{ij}$ thus range from zero to one, while most rumor cascades exhibit multiple emotions. For instance, emotion scores in replies to rumor cascades can be 70% surprise and 30% fear.

We calculate a 4-dimensional score $b_{ij}$ for the bipolar emotion pairs in Plutchik’s wheel of emotions, one for each of the four axes: “anticipation–surprise”, “anger–fear”, “trust–disgust”, and “joy–sadness”. Each of the four bipolar emotion pairs thus measures the difference between an emotion (e. g., joy) and its complement at the opposite side of the wheel (e. g., sadness). We use bipolar emotions due to the strong linear dependence among the eight basic emotions. Adding all basic emotions to the same model would make the estimation rank-deficient. Therefore, we focus on bipolar emotions as these allow for all basic emotions to be examined in the same model.

In Plutchik’s emotion model, emotion scores sum up to one across the basic emotions. We thus omit 149 rumor cascades that do not contain any emotional words from the NRC emotion lexicon (since, otherwise, the denominator is not defined).

### Validation of dictionary approach

Our results rely on the validity of dictionaries to extract sentiment and emotions from online rumors. We thus checked how the perceived sentiment and emotions in rumors align with the lexicon-based sentiment score and emotion scores. For this, we conducted two user studies (see Supplementary Section A), where participants were asked to rate the perceived sentiment, as well as the perceived emotions, in a given rumor. In both studies, the participants exhibited a statistically significant interrater agreement (using Kendall’s *W*). Importantly, we found Spearman’s correlation coefficients for the human labels and the dictionary-based scores to be positive and statistically significant; both for sentiment ($r_s=0.11$, $p<0.01$) and emotions ($r_s=0.13$, $p<0.01$). In sum, the results add to the validity of our lexicon-based approach. The lexicon-based approach should thus capture the perceived sentiment, as well as the perceived emotions, in online rumors.

### Variable description

A rumor cascade $j = 1, \ldots , N$ belonging to rumor *i* is given by a tree structure $T_{ij} = (r_{ij}, t_{ij0}, R_{ij})$ with root tweet $r_{ij}$, the root node’s timestamp $t_{ij0}$, and a set of retweets $R_{ij} = \{ (p_{ijk}, t_{ijk}) \}_{k}$, where each retweet is a 2-tuple comprising a parent $p_{ijk}$ and a timestamp $t_{ijk}$. The root denotes the original sender of the tweet.

#### Cascade structure

Based on the tree structure $T_{ij}$, we compute the following variables $y_{ij}$ characterizing the underlying diffusion dynamics (Fig. 8):

- *Size* The size refers to the overall number of retweets in the cascade, that is, $| R_{ij} | + 1$. Hence, it measures how many users interacted with a tweet.
- *Lifetime* This is the overall timespan during which the tweet travels through the network, defined as $\max {\{ t_{ijk} \}_k} - t_{ij0}$.
- *Structural virality*^39^ This metric measures the trade-off between a cascade that stems from a single retweet and a cascade that has a chain structure, thus quantifying how frequently and how extensively a message is retweeted. Formally, it is defined as the average “distance” between all pairs of retweeters^39^, i. e., $v(T_{ij}) = \frac{1}{n\,(n-1)} \sum \limits _{j_1=1}^{n}\sum \limits _{j_2=1}^{n} d_{ij_1,ij_2}$ for a cascade $T_{ij}$ with *n* nodes and where $d_{ij_1,ij_2}$ is the shortest path between nodes ${ij}_1$ and ${ij}_2$ (similar to the Wiener index).

#### Social influence

Following earlier research^17,24,63^, the social influence of the root $r_{ij}$ is quantified by the following covariates $x_{ij}$:

- *Account age* The age of the root’s account (in years).
- *Out-degree* The number of followers, i. e., the number of accounts that follow the user (in 1000s).
- *In-degree* The number of followees, i. e., the number of accounts whom the user follows (in 1000s).
- *User engagement* For the sender, past engagement is measured by the past number of interactions on Twitter (i. e., tweets, shares, replies, and likes) relative to the account age^17^. Formally, it computes to $(T + R + P + L) / A$ given the past volume of tweets *T*, retweets *R*, replies *P*, and likes *L* divided by the root’s account age *A* (in days).
- *Verified account* A binary dummy indicating whether the account of the root has been officially verified by Twitter ($=1$; otherwise $=0$). This is shown by a blue badge that is reserved for users of public interest (e. g., celebrities, politicians).

All of the above variables are computed at the level of cascades as our unit of analysis. Time is not explicitly included but later captured in the random effects (we also performed a separate analysis with time effects as part of our robustness checks).

### Research framework

In this work, our objective is to attribute differences in the structural properties of true vs. false rumors to positive and negative language as well as words associated with certain emotions. For this purpose, we link the structural properties to the sentiment and emotions conveyed by the language in the replies to rumor cascades. Specifically, we address the following questions: (1) How are variations in language characterized by positive and negative sentiment associated with differences in the structural properties of true vs. false rumor cascades? (2) How does the presence words conveying certain emotions (e. g., anger, trust) explain differences in the structural properties of true vs. false rumor cascades?

Our research questions aim to explain why false rumors (as compared to true rumors) have a longer lifetime, a larger size, and higher structural virality. As defined before, sentiment is a one-dimensional measure along with positive and negative polarity, while emotions refer to a granular, bipolar assessment of arousal along multiple dimensions. In answering the above research questions, we are interested in the marginal effects (that is, by controlling for other sources of heterogeneity).

### Model specification

We specify regression models that explain the cascade structure based on positive and negative language as well as emotional words, while also accounting for further sources of heterogeneity. Recall that the cascade structure (i. e., the lifetime, size, and structural virality) is given by $y_{ij}$. Furthermore, let $\phi _i$ denote the veracity of rumor *i*. Here we define a true rumor as $\phi _i = 0$ and a false rumor as $\phi _i = 1$. Rumors of mixed veracity are included later as part of the robustness checks.

#### Controls

In order to estimate marginal effects, we include several control variables. The control variables are: the social influence of the root $x_{ij}$ (as cascades are likely to diffuse more extensively from influential users) and the veracity $\phi _i$. The latter measures, all else being equal, the relative contribution of veracity to a rumor going viral. In addition, we control for heterogeneity among rumors by using rumor-specific random effects. The latter is important as it accounts for other unobserved factors (e. g., rumor topic, links to external websites, posting date) that may influence the spreading dynamics.

#### Regression

Based on the above, we yield the following hierarchical generalized linear model for our analysis of language classified by positive and negative sentiment:

1 $$\begin{aligned} y_{ij}&= {\beta} _0 + {\beta} _1^T \, x_{ij} + {\beta} _2 \, {\phi} _i + {\beta} _3 \, {s}_{ij} + {\beta} _4 \, ({\phi }_i \times {s}_{ij}) + {u}_i \end{aligned}$$

with intercept $\beta _0$, rumor-specific random effects $u_i$, and coefficients $\beta _1, \ldots , \beta _4$ (out of which $\beta _1$ is a vector). Here the dependent variable is given by $y_{ij}$ (i. e., lifetime, size, or structural virality). Depending on the actual choice of the dependent variable, a different distribution is modeled and, hence, a different estimator must be used. This is detailed later. The notation $(\phi _i \times s_{ij})$ refers to a one-way interaction term.

For our analysis of emotional language, a hierarchical generalized linear model is analogously obtained whereby the sentiment variable $s_{ij}$ is replaced by the bipolar emotions pairs $b_{ij} \in {\mathbb {R}}^4$, i. e.,

2 $$\begin{aligned} y_{ij}&= \beta _0 + \beta _1^T \, x_{ij} + \beta _2 \, \phi _i + \beta _3^T \, b_{ij} + \beta _4^T \, (\phi _i \odot b_{ij}) + u_i \end{aligned}$$

with parameters $\beta _0, \ldots , \beta _4$ (out of which $\beta _1$, $\beta _3$, and $\beta _4$ are vectors and where $\odot$ is the element-wise multiplication).

#### Model coefficients

The estimation results for the parameters $\beta _0, \ldots , \beta _4$ characterize the spread of true vs. false rumors as follows:

- $\beta _1$ is the intercept. It represents the baseline structure for a cascade with average properties.
- $\beta _2$ assesses the overall contribution of veracity to diffusion dynamics (after correcting for different emotions and social influence as in true vs. false rumors). Hence, all else being equal, this parameter quantifies to what extent false rumors last longer, spread more widely, and go more viral as compared to true rumors.
- $\beta _3$ measures how tweets with emotional language link to cascade structures. Estimation results for this coefficient have been discussed elsewhere^18,24,26^ and, for reasons of brevity, are thus omitted from our results section. We note that the influence directly attributed to emotional language is consistent with previous research.

Of particular interest is the following parameter:

- $\beta _4$ estimates the relative differences in how emotional language is received in relation to true vs. false rumors. This is captured by the one-way interaction between the emotion variables and veracity. Hence, a positive $\beta _4$ indicates that an increase in the fraction of emotional words of a certain category is associated with larger increases of the dependent variable for false vs. true rumors. As we controlled for other sources of heterogeneity, these estimates are “ceteris paribus,” that is, all else being equal, they measure how much larger/smaller is the effect of language classified by emotions on size, lifetime, and structural virality if the rumor is false.

### Estimation details

The actual choice of the dependent variable requires a different estimator in order to account for the underlying distribution. Cascade size represents count data and its variance is larger than its mean. We thus adjust for overdispersion and apply negative binomial regression with log-transformation. For lifetime, prior research has suggested that response times are log-normally distributed^63^. Accordingly, we log-transform the lifetimes. Results of the Shapiro-Wilk test for normality applied to the log-transformed variable suggest that the null hypothesis of normal distribution cannot be rejected. This allows us to estimate the model using ordinary least squares (OLS). For structural virality, we use a gamma regression with log-link, which is a common choice for modeling positively skewed, non-negative continuous variables.

Our implementation uses the lme4 package in R 3.6.3. This ensures that random effects are considered. Approaches for winsorizing or censoring the data (or other filtering options) were intentionally disregarded, as we consider all observations to be informative, especially those in the tails. We nevertheless performed a robustness check with winsorizing, yielding consistent outcomes. We *z*-standardized all variables in order to facilitate interpretability. Accordingly, the regression coefficients measure the relationship with the dependent variable in standard deviations.

### Robustness checks

We conducted the following checks to validate the robustness of our results.

#### Fine-grained emotions

Instead of having four bipolar dimensions, we ran a regression with all eight fine-grained emotions as separate variables (see Supplementary Tables S3–S5). Consistent with our previous findings, we again find that words associated with emotions like anticipation, trust, and anger accelerate the spread of false rumors. However, the estimation is rank-deficient and, hence, our main analysis is instead based on bipolar emotion pairs.

#### Additional checks

We conducted additional checks to validate the robustness of our results: (1) we ran separate regressions for true vs. false rumors. (2) to ensure robustness across the complete time period of the study, we used clustered standard errors at the annual level and repeated the analysis for different time periods. Furthermore, we included dummy variables for each year in our sample to control for year-level effects. The results show a good agreement of the coefficients of all variables and support the robustness of our results across time periods (see Supplementary Section B). (3) The validity of our estimates was ensured by following common practice for regression modeling. In particular, we determined the variance inflation factor (VIF) to be below the critical threshold of five^64^. (4) We added non-linear regressors (i. e., quadratic terms) for each emotion to our regression models. In all cases, our results are robust consistently support our findings. (5) We analyzed how the diversity of emotion scores is association with the spread of rumors. Here we extended our regression models with a variable that measures the sum of squares over the 8-dimensional vector comprising the different emotion scores. We find that a higher diversity of emotion scores is associated with higher values for cascade size, duration, and structural virality (see Supplementary Section B).

## Supplementary information

Supplementary Information.

## Data Availability

Permission from Twitter to analyze the dataset was obtained. All data needed to evaluate the conclusions in the paper are publicly available (and the source reported in the paper). Replication code for this study is available via https://github.com/DominikBaer95/Emotions_true_vs_false_online_rumors.
