# Supplementary material for: Emotions explain differences in the diffusion of true vs. false social media rumors
Source: Sci Rep. 2021 Nov 22;11:22721. doi: 10.1038/s41598-021-01813-2 (PMC8608927; doi:10.1038/s41598-021-01813-2)
Supplement: Supplementary file 1 — Supplementary Information. [file 41598_2021_1813_MOESM1_ESM.pdf]

Supplementary Materials

Emotions Explain Differences in the Diffusion of True vs. False Social Media Rumors

Nicolas Pröllochs,*[1] Dominik Bär,[2] Stefan Feuerriegel,[2,3]

[1]JLU Giessen, Giessen, 35394, Germany
[2]LMU Munich, Munich, 80539, Germany
[3]ETH Zurich, Zurich, 8092, Switzerland

*E-mail: nicolas.proellochs@wi.jlug.de.

Tables S1 – S5

Table S1: Regression results for sentiment. The dependent variables are cascade size (column 1), cascade duration (column 2), and structural virality (column 3). Rumor-specific random effects are included.

	**Size**	**Duration**	**Virality**
Intercept	3.710^{***}	4.001^{***}	0.742^{***}
	(0.101)	(0.109)	(0.015)
Falsehood	0.006	-0.305^{*}	-0.021
	(0.110)	(0.119)	(0.016)
User Engagement	-0.184^{***}	-0.587^{***}	0.035^{***}
	(0.027)	(0.036)	(0.005)
Verified Account	0.848^{***}	0.754^{***}	0.156^{***}
	(0.056)	(0.063)	(0.009)
Account Age	-0.180^{***}	-0.232^{***}	-0.003
	(0.015)	(0.018)	(0.002)
Followers	0.755^{***}	0.235^{***}	0.002
	(0.041)	(0.016)	(0.002)
Followees	0.227^{***}	0.131^{***}	0.017^{***}
	(0.022)	(0.016)	(0.002)
Sentiment	-0.165^{***}	-0.100^{*}	-0.014^{**}
	(0.040)	(0.042)	(0.005)
Falsehood $\times$ Sentiment	0.479^{***}	0.319^{***}	0.047^{***}
	(0.044)	(0.047)	(0.006)
Rumor-Specific Random Effects	Yes	Yes	Yes
AIC	182,185	71,979	32,108

$^{***}p < 0.001$; $^{**}p < 0.01$; $^{*}p < 0.05$

Table S2: Regression results for bipolar emotion pairs. The dependent variables are cascade size (column 1), cascade duration (column 2), and structural virality (column 3). Rumor-specific random effects are included.

	**Size**	**Duration**	**Virality**
Intercept	3.725***	4.023***	0.746***
	(0.101)	(0.110)	(0.015)
Falsehood	−0.007	−0.327**	−0.025
	(0.110)	(0.120)	(0.016)
User Engagement	−0.188***	−0.588***	0.035***
	(0.027)	(0.036)	(0.005)
Verified Account	0.840***	0.751***	0.155***
	(0.056)	(0.063)	(0.009)
Account Age	−0.179***	−0.232***	−0.003
	(0.015)	(0.018)	(0.002)
Followers	0.751***	0.235***	0.002
	(0.041)	(0.016)	(0.002)
Followees	0.223***	0.130***	0.017***
	(0.022)	(0.016)	(0.002)
JoySadness	0.000	−0.006	0.000
	(0.033)	(0.036)	(0.004)
TrustDisgust	−0.189***	−0.051	−0.007
	(0.043)	(0.045)	(0.005)
AngerFear	−0.020	−0.080	−0.004
	(0.046)	(0.047)	(0.005)
AnticipationSurprise	−0.105*	−0.131*	−0.023***
	(0.047)	(0.056)	(0.006)
Falsehood × JoySadness	0.013	0.047	0.001
	(0.039)	(0.041)	(0.005)
Falsehood × TrustDisgust	0.402***	0.205***	0.031***
	(0.047)	(0.049)	(0.006)
Falsehood × AngerFear	0.212***	0.200***	0.020***
	(0.050)	(0.052)	(0.006)
Falsehood × AnticipationSurprise	0.339***	0.285***	0.051***
	(0.050)	(0.060)	(0.007)
Rumor-Specific Random Effects	Yes	Yes	Yes
AIC	182,147	72,013	32,072

$^{***}p < 0.001$; $^{**}p < 0.01$; $^{*}p < 0.05$

Table S3: Regression results with basic emotions. The dependent variable is cascade size. We fit separate regression models, each including one of the 8 basic emotions. This estimation procedure is adopted because basic emotions sum to 1 and are thus subject to a linear dependency. Rumor-specific random effects are included.

	(1)	(2)	(3)	(4)	(5)	(6)	(7)	(8)
Intercept	3.610^{***}	3.604^{***}	3.621^{***}	3.637^{***}	3.635^{***}	3.608^{***}	3.618^{***}	3.662^{***}
	(0.100)	(0.004)	(0.100)	(0.100)	(0.101)	(0.100)	(0.100)	(0.092)
Falsehood	0.136	0.149^{***}	0.131	0.101	0.107	0.144	0.126	0.085
	(0.109)	(0.004)	(0.110)	(0.109)	(0.110)	(0.109)	(0.109)	(0.101)
User Engagement	-0.197^{***}	-0.202^{***}	-0.203^{***}	-0.197^{***}	-0.198^{***}	-0.202^{***}	-0.197^{***}	-0.201^{***}
	(0.027)	(0.004)	(0.027)	(0.027)	(0.027)	(0.027)	(0.027)	(0.031)
Verified Account	0.896^{***}	0.896^{***}	0.896^{***}	0.872^{***}	0.879^{***}	0.894^{***}	0.894^{***}	0.894^{***}
	(0.056)	(0.004)	(0.056)	(0.056)	(0.056)	(0.056)	(0.056)	(0.055)
Account Age	-0.172^{***}	-0.178^{***}	-0.179^{***}	-0.182^{***}	-0.177^{***}	-0.178^{***}	-0.179^{***}	-0.180^{***}
	(0.015)	(0.004)	(0.015)	(0.015)	(0.015)	(0.015)	(0.015)	(0.015)
Followers	0.766^{***}	0.774^{***}	0.770^{***}	0.767^{***}	0.763^{***}	0.774^{***}	0.773^{***}	0.771^{***}
	(0.041)	(0.004)	(0.041)	(0.041)	(0.041)	(0.041)	(0.041)	(0.014)
Followees	0.235^{***}	0.242^{***}	0.242^{***}	0.239^{***}	0.231^{***}	0.242^{***}	0.242^{***}	0.244^{***}
	(0.022)	(0.004)	(0.022)	(0.022)	(0.022)	(0.022)	(0.022)	(0.014)
Anger	0.014							
	(0.039)							
Falsehood × Anger	0.181^{***}							
	(0.044)							
Fear		-0.039^{***}						
		(0.004)						
Falsehood × Fear		-0.011^{**}						
		(0.004)						
Anticipation			-0.072^{*}					
			(0.035)					
Falsehood × Anticipation			0.116^{**}					
			(0.041)					
Trust				-0.076^{*}				
				(0.031)				
Falsehood × Trust				0.313^{***}				
				(0.038)				
Surprise					0.083			
					(0.069)			
Falsehood × Surprise					-0.286^{***}			
					(0.071)			
Sadness						−0.026		
						(0.033)		
Falsehood × Sadness						0.141^{***}		
						(0.039)		
Joy							−0.041	
							(0.031)	
Falsehood × Joy							0.165^{***}	
							(0.038)	
Disgust								0.244^{***}
								(0.036)
Falsehood × Disgust								-0.333^{***}
								(0.039)
Rumor-Specific Random Effects	Yes	Yes	Yes	Yes	Yes	Yes	Yes	Yes
AIC	182,393	182,484	182,484	182,359	182,337	182,462	182,458	182,449

$^{***}p < 0.001$; $^{**}p < 0.01$; $^{*}p < 0.05$

Table S4: Regression results with basic emotions. The dependent variable is cascade lifetime. We fit separate regression models, each including one of the 8 basic emotions. This estimation procedure is adopted because basic emotions sum to 1 and are thus subject to a linear dependency. Rumor-specific random effects are included.

	(1)	(2)	(3)	(4)	(5)	(6)	(7)	(8)
Intercept	3.946***	3.946***	3.957***	3.955***	3.982***	3.941***	3.944***	3.952***
	(0.108)	(0.108)	(0.109)	(0.108)	(0.110)	(0.108)	(0.108)	(0.109)
Falsehood	−0.224	−0.221	−0.233*	−0.244*	−0.264*	−0.217	−0.227	−0.232
	(0.118)	(0.118)	(0.119)	(0.118)	(0.119)	(0.118)	(0.118)	(0.119)
User Engagement	−0.590***	−0.591***	−0.591***	−0.590***	−0.589***	−0.590***	−0.588***	−0.589***
	(0.036)	(0.036)	(0.036)	(0.036)	(0.036)	(0.036)	(0.036)	(0.036)
Verified Account	0.779***	0.778***	0.779***	0.771***	0.771***	0.779***	0.777***	0.779***
	(0.063)	(0.063)	(0.063)	(0.063)	(0.063)	(0.063)	(0.063)	(0.063)
Account Age	−0.229***	−0.229***	−0.230***	−0.231***	−0.229***	−0.229***	−0.229***	−0.230***
	(0.018)	(0.018)	(0.018)	(0.018)	(0.018)	(0.018)	(0.018)	(0.018)
Followers	0.236***	0.236***	0.236***	0.236***	0.235***	0.236***	0.236***	0.236***
	(0.016)	(0.016)	(0.016)	(0.016)	(0.016)	(0.016)	(0.016)	(0.016)
Followees	0.134***	0.135***	0.135***	0.134***	0.133***	0.135***	0.136***	0.135***
	(0.016)	(0.016)	(0.016)	(0.016)	(0.016)	(0.016)	(0.016)	(0.016)
Anger	−0.045							
	(0.039)							
Falsehood × Anger	0.111*							
	(0.044)							
Fear		0.038						
		(0.057)						
Falsehood × Fear		−0.086						
		(0.060)						
Anticipation			−0.051					
			(0.041)					
Falsehood × Anticipation			0.077					
			(0.046)					
Trust				−0.036				
				(0.035)				
Falsehood × Trust				0.202***				
				(0.040)				
Surprise					0.140			
					(0.073)			
Falsehood × Surprise					−0.270***			
					(0.075)			
Sadness						0.019		
						(0.037)		
Falsehood × Sadness						0.033		
						(0.042)		
Joy							0.005	
							(0.036)	
Falsehood × Joy							0.100*	
							(0.041)	
Disgust								0.039
								(0.051)
Falsehood × Disgust								−0.096
								(0.055)
Rumor-Specific Random Effects	Yes	Yes	Yes	Yes	Yes	Yes	Yes	Yes
AIC	72,092	72,097	72,101	72,033	72,051	72,097	72,079	72,095

$^{***}p < 0.001$; $^{**}p < 0.01$; $^{*}p < 0.05$

Table S5: Regression results with basic emotions. The dependent variable is structural virality. We fit separate regression models, each including one of the 8 basic emotions. This estimation procedure is adopted because basic emotions sum to 1 and are thus subject to a linear dependency. Rumor-specific random effects are included.

	(1)	(2)	(3)	(4)	(5)	(6)	(7)	(8)
Intercept	0.716***	0.715***	0.719***	0.716***	0.720***	0.715***	0.715***	0.719***
	(0.018)	(0.018)	(0.018)	(0.018)	(0.018)	(0.018)	(0.018)	(0.018)
Falsehood	0.016	0.017	0.012	0.014	0.010	0.016	0.015	0.012
	(0.020)	(0.020)	(0.020)	(0.020)	(0.020)	(0.020)	(0.020)	(0.020)
User Engagement	0.034***	0.034***	0.034***	0.034***	0.034***	0.034***	0.035***	0.034***
	(0.005)	(0.005)	(0.005)	(0.005)	(0.005)	(0.005)	(0.005)	(0.005)
Verified Account	0.167***	0.166***	0.166***	0.165***	0.164***	0.166***	0.166***	0.166***
	(0.009)	(0.009)	(0.009)	(0.009)	(0.009)	(0.009)	(0.009)	(0.009)
Account Age	−0.001	−0.001	−0.001	−0.002	−0.001	−0.001	−0.001	−0.002
	(0.002)	(0.002)	(0.002)	(0.002)	(0.002)	(0.002)	(0.002)	(0.002)
Followers	0.002	0.002	0.002	0.002	0.002	0.002	0.002	0.002
	(0.002)	(0.002)	(0.002)	(0.002)	(0.002)	(0.002)	(0.002)	(0.002)
Followees	0.016***	0.016***	0.016***	0.016***	0.016***	0.016***	0.017***	0.016***
	(0.002)	(0.002)	(0.002)	(0.002)	(0.002)	(0.002)	(0.002)	(0.002)
Anger	−0.005							
	(0.004)							
Falsehood × Anger	0.013**							
	(0.005)							
Fear		−0.007						
		(0.006)						
Falsehood × Fear		0.003						
		(0.006)						
Anticipation			−0.014**					
			(0.004)					
Falsehood × Anticipation			0.020***					
			(0.005)					
Trust				0.000				
				(0.004)				
Falsehood × Trust				0.022***				
				(0.005)				
Surprise					0.017*			
					(0.008)			
Falsehood × Surprise					−0.041***			
					(0.009)			
Sadness						0.003		
						(0.004)		
Falsehood × Sadness						0.015**		
						(0.005)		
Joy							0.002	
							(0.004)	
Falsehood × Joy							0.014**	
							(0.005)	
Disgust								0.013*
								(0.006)
Falsehood × Disgust								−0.022***
								(0.006)
Rumor-Specific Random Effects	Yes	Yes	Yes	Yes	Yes	Yes	Yes	Yes
AIC	32,267	32,274	32,263	32,194	32,158	32,222	32,233	32,259

$^{***}p < 0.001$; $^{**}p < 0.01$; $^{*}p < 0.05$

A User Studies to Validate Dictionary Approach

Our results rely on the validity of dictionaries to extract emotions from online rumors. We thus check how (i) perceived sentiment aligns with the lexicon-based sentiment and (ii) how perceived emotions align with the lexicon-based emotion scores. For this, we conducted two user studies using the online survey platform Prolific (`https://www.prolific.co/`). For both studies, we randomly sampled 100 rumors from Twitter and presented them to $n = 7$ participants (English native speakers).

The participants of the first study were asked to rate the sentiment conveyed in each tweet on a Likert scale from -3 to $+3$ (here: -3 indicates negative sentiment, while $+3$ refers to a positive sentiment). The participants exhibited a statistically significant interrater agreement according to Kendall's W ($p < 0.01$). Furthermore, we compute the correlation between the human labels and the lexicon-based sentiment score. We found Spearman's correlation coefficient to be positive ($r_s = 0.11$) and statistically significant ($p < 0.01$). In sum, the results add to the validity of our lexicon-based approach. The lexicon-based approach should thus largely match the perceived sentiment in online rumors.

In the second study, the participants were instructed to rate the presence of the eight basic emotions on a Likert scale from -3 to $+3$ (here: -3 indicates no emotion present while $+3$ refers to a high degree of emotion present) for each tweet. As shown in the following table, the participants exhibited a statistically significant interrater agreement according to Kendall's W for each of the 8 basic emotions ($p < 0.01$). Overall, the correlation between the dictionary-based emotion scores and human annotations is $r_s = 0.13$ ($p < 0.01$) and thus statistically significant at common significance thresholds. This demonstrates that our dictionary approach is able to capture emotions in online rumors.

Anger	Anticipation	Disgust	Fear	Joy	Sadness	Surprise	Trust
0.474***	0.198***	0.427***	0.406***	0.364***	0.408***	0.227***	0.230***

Kendall's W coefficient for the interrater agreement between survey participants.
$^{*}p < 0.1$, $^{**}p < 0.05$, $^{***}p < 0.01$.

B Additional Robustness Checks

B.1 Analysis of Annual Effects

Our sample contains a comprehensive set of fact-checked rumors from Twitter during the time period from its founding in 2006 through 2017. We validate the robustness of our results with respect to different time periods by incorporating dummy variables for each year of our observation period. The estimation results with year-level effects are shown in Table S6 and Table S7. The coefficients of all variables are in good agreement and support the robustness of our results across time periods. These findings suggest that the observed effects of language classified by (i) sentiment and (ii) emotions on size, lifetime, and structural virality should generalize well.

Table S6: Regression results for sentiment. The dependent variables are cascade size (column 1), cascade duration (column 2), and structural virality (column 3). Rumor-specific random effects are included. Year-level effects are included.

	Size	Duration	Virality
Intercept	−0.279	0.673	−0.068
	(1.304)	(0.965)	(0.330)
Falsehood	0.032	−0.308**	−0.020
	(0.107)	(0.118)	(0.016)
User Engagement	−0.218***	−0.598***	0.029***
	(0.027)	(0.036)	(0.005)
Verified Account	0.901***	0.812***	0.161***
	(0.056)	(0.063)	(0.009)
Account Age	−0.194***	−0.247***	−0.005*
	(0.015)	(0.018)	(0.002)
Followers	0.715***	0.228***	0.001
	(0.040)	(0.016)	(0.002)
Followees	0.209***	0.128***	0.016***
	(0.022)	(0.016)	(0.002)
Sentiment	−0.159***	−0.094*	−0.014**
	(0.040)	(0.042)	(0.005)
Falsehood × Sentiment	0.459***	0.304***	0.046***
	(0.044)	(0.046)	(0.006)
Rumor-Specific Random Effects	Yes	Yes	Yes
Year-Level Effects	Yes	Yes	Yes
AIC	181,873	71,806	32,003

$^{***}p < 0.001$; $^{**}p < 0.01$; $^{*}p < 0.05$

Table S7: Regression results for bipolar emotion pairs. The dependent variables are cascade size (column 1), cascade duration (column 2), and structural virality (column 3). Rumor-specific random effects are included. Year-level effects are included.

	Size	Duration	Virality
Intercept	-0.249	0.683	-0.079
	(2.163)	(0.965)	(0.309)
Falsehood	0.025	-0.329^{**}	-0.002
	(0.099)	(0.119)	(0.020)
User Engagement	-0.220^{***}	-0.599^{***}	0.030^{***}
	(0.030)	(0.036)	(0.005)
Verified Account	0.897^{***}	0.809^{***}	0.165^{***}
	(0.054)	(0.063)	(0.009)
Account Age	-0.193^{***}	-0.247^{***}	-0.004
	(0.015)	(0.018)	(0.002)
Followers	0.708^{***}	0.228^{***}	0.001
	(0.014)	(0.016)	(0.002)
Followees	0.206^{***}	0.127^{***}	0.015^{***}
	(0.014)	(0.016)	(0.002)
JoySadness	-0.003	-0.005	0.000
	(0.026)	(0.036)	(0.004)
TrustDisgust	-0.177^{***}	-0.047	-0.007
	(0.032)	(0.045)	(0.005)
AngerFear	-0.018	-0.079	-0.005
	(0.032)	(0.047)	(0.005)
AnticipationSurprise	-0.106^{**}	-0.125^{*}	-0.023^{***}
	(0.037)	(0.056)	(0.006)
Falsehood × JoySadness	0.019	0.038	0.000
	(0.030)	(0.041)	(0.005)
Falsehood × TrustDisgust	0.384^{***}	0.195^{***}	0.029^{***}
	(0.035)	(0.049)	(0.006)
Falsehood × AngerFear	0.194^{***}	0.196^{***}	0.019^{**}
	(0.035)	(0.051)	(0.006)
Falsehood × AnticipationSurprise	0.327^{***}	0.275^{***}	0.049^{***}
	(0.040)	(0.059)	(0.006)
Rumor-Specific Random Effects	Yes	Yes	Yes
Year-Level Effects	Yes	Yes	Yes
AIC	181,284	71,839	31,958

$^{***}p < 0.001$; $^{**}p < 0.01$; $^{*}p < 0.05$

Analysis of Emotional Uniformity

We calculate the sum of squares over the 8-dimensional vector comprising the different emotion scores. This metric provides a measure of emotional uniformity by attributing higher scores to rumors that have lower diversity among emotion classifications. For instance, if a rumor contains words associated with only one emotion (e. g., anger) and no other emotion, then it receives a high score. In contrast, a lower score is assigned if language is associated with all emotions to a similar extent. The regression estimates are shown in Table S8. We find that a higher level of uniformity across emotion scores (i. e., lower diversity) is associated with smaller values for cascade size, duration, and structural virality.

Table S8: Regression results for emotional uniformity. The dependent variables are cascade size (column 1), cascade duration (column 2), and structural virality (column 3). Rumor-specific random effects are included.

	**Size**	**Duration**	**Virality**
Intercept	3.498***	3.837***	0.719***
	(0.098)	(0.107)	(0.014)
Falsehood	0.162	−0.198	−0.006
	(0.107)	(0.116)	(0.016)
User Engagement	−0.171***	−0.575***	0.036***
	(0.027)	(0.036)	(0.005)
Verified Account	0.793***	0.700***	0.146***
	(0.056)	(0.062)	(0.009)
Account Age	−0.179***	−0.235***	−0.003
	(0.015)	(0.018)	(0.002)
Followers	0.740***	0.234***	0.001
	(0.040)	(0.016)	(0.002)
Followees	0.218***	0.129***	0.016***
	(0.021)	(0.015)	(0.002)
Emotional Uniformity	−0.355***	−0.327***	−0.052***
	(0.012)	(0.018)	(0.002)
Rumor-Specific Random Effects	Yes	Yes	Yes
AIC	181,781	71,767	31,673

***$p < 0.001$; **$p < 0.01$; *$p < 0.05$

B.2 Analysis of Rumors With Mixed Veracity

In our main analysis, we focused on rumors that can clearly be determined as "true" or "false." However, some rumors were categorized as being of "mixed" veracity by the fact-checking organizations (where the veracity could not be clearly designated as true or false). As an exploratory analysis, we analyze differences in the spreading dynamics of mixed rumors. Due to data limitations, we re-estimate our model for a smaller set of independent variables, i. e., only the control variables. The regression results for cascade size, cascade duration, and cascade virality are shown in Table S9. Compared to true rumors, we find that rumor cascades of mixed veracity exhibit a smaller cascade size and virality but spread over a longer time horizon.

Table S9: Regression results for mixed rumors. The dependent variables are cascade size (column 1), cascade duration (column 2), and structural virality (column 3).

	**Size**	**Duration**	**Virality**
Intercept	1.662***	1.732***	0.177***
	(0.012)	(0.028)	(0.002)
Falsehood	1.240***	0.285***	0.058***
	(0.013)	(0.031)	(0.003)
Mixed	−0.096***	0.200***	−0.021***
	(0.017)	(0.043)	(0.003)
User Engagement	−0.193***	−0.262***	0.077***
	(0.005)	(0.008)	(0.001)
Verified Account	1.553***	1.753***	0.439***
	(0.036)	(0.049)	(0.007)
Account Age	−0.147***	−0.177***	0.014***
	(0.005)	(0.010)	(0.001)
Followers	2.940***	0.168***	0.030***
	(0.005)	(0.006)	(0.001)
Followees	0.567***	0.195***	0.089***
	(0.005)	(0.007)	(0.001)
Rumor-Specific Random Effects	No	No	No
AIC	761,187	182,629	86,614

$^{***}p < 0.001$; $^{**}p < 0.01$; $^{*}p < 0.05$
